# Exploring the Relationships Between Hemodynamic Stresses in the Carotid Arteries

**DOI:** 10.3389/fcvm.2020.617755

**Published:** 2021-02-03

**Authors:** Magnus Ziegler, Jesper Alfraeus, Elin Good, Jan Engvall, Ebo de Muinck, Petter Dyverfeldt

**Affiliations:** ^1^Division of Cardiovascular Medicine, Department of Health, Medicine and Caring Sciences, Linköping University, Linköping, Sweden; ^2^Center for Medical Image Science and Visualization (CMIV), Linköping University, Linköping, Sweden; ^3^Department of Cardiology, Department of Health, Medicine and Caring Sciences, Linköping University, Linköping, Sweden; ^4^Department of Clinical Physiology, Department of Health, Medicine and Caring Sciences, Linköping University, Linköping, Sweden

**Keywords:** carotid bifurcation, magnetic resonnance imaging (MRI), turbulence, wall shear stress (WSS), atherosclerosis

## Abstract

**Background:** Atherosclerosis manifests as a focal disease, often affecting areas with complex hemodynamics such as the carotid bifurcation. The magnitude and regularity of the hemodynamic shear stresses acting on the vessel wall are thought to generate risk patterns unique to each patient and play a role in the pathogenesis of atherosclerosis. The involvement of different expressions of shear stress in the pathogenesis of carotid atherosclerosis highlights the need to characterize and compare the differential impact of the various expressions of shear stress in the atherosclerotic carotid bifurcation. Therefore, the aim of this study is to characterize and compare hemodynamic wall shear stresses (WSS) in the carotid arteries of subjects with asymptomatic atherosclerotic plaques. Shear stresses were also compared against vessel diameter and bifurcation angle to examine the relationships with the geometry of the carotid bifurcation.

**Methods:** 4D Flow MRI and contrast-enhanced MRA data were acquired for 245 subjects with atherosclerotic plaques of at least 2.7 mm in conjunction with the Swedish CArdioPulmonary bioImage Study (SCAPIS). Following automatic segmentation and geometric analysis, time-resolved WSS and near-wall turbulent kinetic energy (nwTKE) were derived from the 4D Flow data. Whole-cycle parameters including time-averaged WSS and nwTKE, and the oscillatory shear index (OSI) were calculated. Pairwise Spearman rank-correlation analyses were used to investigate relationships among the hemodynamic as well as geometric parameters.

**Results:** One hundred and seventy nine subjects were successfully segmented using automated tools and subsequently geometric and hemodynamic analyses were performed. Temporally resolved WSS and nwTKE were strongly correlated, ρ = 0.64. Cycle-averaged WSS and nwTKE were moderately correlated, ρ = 0.57. Cycle-average nwTKE was weakly correlated to OSI (ρ = −0.273), revealing that nwTKE provides information about disturbed flow on the vessel wall that OSI does not. In this cohort, there was large inter-individual variation for both WSS and nwTKE. Both WSS and nwTKE varied most within the external carotid artery. WSS, nwTKE, and OSI were weakly correlated to vessel diameter and bifurcation angle.

**Conclusion:** The turbulent and mean component of WSS were examined together *in vivo* for the first time, and a strong correlation was found between them. nwTKE presents the opportunity to quantify turbulent wall stresses *in vivo* and gain insight into the effects of disturbed flow on the vessel wall. Neither vessel diameter nor bifurcation angle were found to be strongly correlated to the turbulent or mean component of WSS in this cohort.

## Introduction

Atherosclerosis manifests as a focal disease, predominantly affecting areas with complex hemodynamics such as the outer walls of vessel bifurcations, recirculation zones, and areas of stasis (1–3). The carotid bifurcation is one such focal point, and emboli originating from atherosclerotic plaques in this region are responsible for up to one quarter of all strokes (4). The potential relationship between carotid hemodynamics and stroke highlights the need for studies of carotid hemodynamics in patients with carotid atherosclerosis.

Complex hemodynamics give rise to different expressions of shear stresses that act on the vessel wall. Different expressions of shear stress are believed to be involved in different stages of the pathogenesis of atherosclerosis (5). Regions with consistently low WSS have been associated with the early development of atherosclerosis, and regions with low WSS have also been found to have larger lesions with a more vulnerable plaque phenotype (3, 5–8). High WSS, on the other hand, is thought to have atheroprotective effects (1). Similar to low WSS, regions with elevated oscillatory shear index (OSI) have been suggested to play a role in early atherosclerosis. The OSI reflects directional variations in WSS over the cardiac cycle but is sometimes misinterpreted as representing the chaotic aspects of WSS. However, WSS can also be separated into the mean WSS, which is the aspect of WSS typically investigated, and turbulent WSS (tWSS), which reflects the chaotic nature of WSS in disturbed flows (9). The role played by disturbed and turbulent flows in atherosclerosis is not yet fully understood, but turbulence has been associated with increased endothelial cell turnover *in-vitro*, and may trigger plaque rupture through erosion of the plaque's fibrous cap (10–14). Interestingly, disturbed and turbulent flows may also play a crucial role in the erosion and subsequent thrombotic complications in the less vulnerable type of plaque that intensive statin treatment can create (15).

Comprehensive characterization of WSS in the carotid bifurcation is afforded by methods based on time-resolved three-dimensional MR flow imaging (4D Flow MRI) (16–20). Investigations leveraging these methods have reported initial data on the degree of WSS in the healthy carotid bifurcation, and those with moderate stenoses (16, 18, 19, 21–31). However, those previous studies have been limited to small cohorts and were primarily focused on the mean aspect of WSS. Consequently, turbulent and disturbed flow in the carotid bifurcation has received less investigative effort, though recent effort has enabled the measurement of near wall turbulent kinetic energy (nwTKE) in 4D Flow MRI data for estimating tWSS *in vivo* (20). The involvement of different expressions of shear stress in the pathogenesis of carotid atherosclerosis highlights the need to characterize and compare the differential impact of the various expressions of shear stress in the atherosclerotic carotid bifurcation *in vivo*. Therefore, the primary aim of this study to use 4D Flow MRI to characterize and compare WSS, OSI, and nwTKE in the carotid bifurcation in a large cohort of subjects with carotid atherosclerosis.

The geometric risk hypothesis for atherosclerosis assumes a direct relationship between exposure to disturbed flow and the geometry of the vessel (1, 32). Unfortunately, at present, there is little *in vivo* data available to examine the relationship between disturbed flow and vessel geometry. In particular, the vessel diameter and the bifurcation angle of the carotid arteries are pointed to as potential surrogate markers of disturbed flow. Therefore, the secondary aim of this study was to examine the relationships between WSS, OSI, and nwTKE and the geometry of the carotid bifurcation and determine if these geometric factors can be used for prediction.

## Materials and Methods

### Study Participants

Subjects were recruited as part of the Swedish CArdioPulmonary bioImage Study (SCAPIS) (33). Two hundred and forty five subjects between 50 and 65 years old with at least one asymptomatic carotid plaque larger than 2.7 mm (revised criteria when compared to original SCAPIS design paper), as measured by ultrasound, were enrolled. Demographics are described in Table 1. This study received ethical approval and all participants gave written, informed consent.

**Table 1:** Cohort mean characteristics for clinical and geometric parameters (*n* = 179).

Age [Years]	59.0 ± 4.1
Gender	Female: 48 (27%) Male: 131 (73%)
Height [cm]	175.4 ± 8.6
Weight [kg]	83.7 ± 13.6
BMI[kg/m^2^]	27.2 ± 3.8
Blood Pressure, Systolic [mmHg]	138 ± 19
Blood Pressure, Diastolic [mmHg]	85 ± 10
Pulse [bpm]	62 ± 10
Smoker	Never: 75 (41%) Former: 78 (43%) Sometimes: 12 (7%) Regular: 11 (6%) Unknown: 3 (2%)
Diabetes	15 (8%)
Carotid Plaques (per side)	Min: 0 Median ± IQR: 1 ± 1 Max: 5
Carotid Plaques (per subject)	Min: 1 Median ± IQR: 2 ± 2 Max: 8
Coronary Calcium Score (Total) [-]	179 ± 404
Diameter CCA [mm]	8.7 ± 1.1
Diameter ICA [mm]	7.5 ± 1.4
Diameter ECA [mm]	5.7 ± 1.0
Bifurcation Angle [^°^]	49.5 ± 13.4

*Values presented as mean ± standard deviation unless otherwise stated*.

### Magnetic Resonance Imaging

MRI data was acquired with a 3T Philips Ingenia scanner (Philips Healthcare, Best, the Netherlands) using an 8-channel dedicated carotid coil (Shanghai Chenguang Medical Technologies, Shanghai, China).

#### Contrast Enhanced MR Angiography

Contrast-enhanced MR angiography (CE-MRA) data was acquired post injection of a gadolinium-based contrast agent (Gadovist, Bayer Schering Pharma AG) to generate bright-blood images for automated segmentations of the vessel lumen and basic geometric analyses. Scan parameters included: a coronal slab with 3D field of view = 200 × 200 × 50 mm^3^ and matrix size 512 × 512 × 100 set to cover the carotid arteries from the clavicle to the circle of Willis, flip angle 27°, echo time 1.8 ms, repetition time 4.9 ms, parallel imaging (SENSE) factor 2, and reconstructed spatial resolution of 0.48 × 0.48 × 0.50 mm^3^.

#### 4D Flow MRI

4D Flow MRI data was acquired using a free-breathing, retrospectively cardiac-gated sequence to measure the hemodynamics of the carotid arteries, based on consensus guidelines but adapted for the carotid arteries (34). The 4D Flow MRI scan was set up as a coronal slab with a 3D field-of-view of 210 × 210 × 23 mm^3^ and matrix size 192 × 192 × 19 set to cover the carotid arteries from the clavicle to the Circle of Willis, resulting in an acquired spatial resolution of 1.1 × 1.1 × 1.2 mm^3^. The data was acquired with an approximately isotropic spatial resolution was used to avoid directionally dependent results, as WSS and nwTKE measurements are dependent on spatial resolution. Additional parameters included: flip angle 10°, echo time 3.5 ms, and velocity encoding range (VENC) 1.2 m/s. VENC was chosen to avoid velocity aliasing and maintain optimum sensitivity for the measurement of TKE. SENSE parallel imaging was used with in-plane and out-of-plane acceleration factors of 1.6. Repetition time was 6.1 ms and two k-space lines were acquired per cardiac cycle, resulting in an acquired temporal resolution of 49 ms. Data was reconstructed to 40 timeframes using a temporal sliding window approach. Total scan time was 8 min. Data was corrected for concomitant gradient field effects on the scanner, while phase-wrapping and background phase-offset errors were corrected offline. Background phase-offset errors were corrected using a weighted 4th order fit to static tissue (35).

### Analysis

#### Segmentation

CE-MRA images were automatically segmented to produce masks of the common carotid artery (CCA), external carotid artery (ECA), and internal carotid artery (ICA) using a 3D convolutional neural network (CNN), built upon on the DeepMedic framework (36, 37). Masks were visually inspected, and if necessary corrected, by an observer (M.Z.) with 5 years of vascular MRI experience using ITK-SNAP. The DeepMedic network (v0.7.0) was implemented in Python 3.6.2 using Tensorflow v1.9.0, and MATLAB 2018a was used for post-processing.

#### Geometric

The automatically generated segmentations of the CCA, ECA, and ICA were additionally used to extract geometric descriptions of the carotid bifurcation. For each bifurcation, the mean diameter of each branch and the bifurcation angle were calculated. The bifurcation angle was calculated using vectors aligned with the centrelines of the ICA and ECA. Both vectors originated at the centreline point nearest to the bifurcation on each branch and intersected the midpoint of the centreline for each branch. Geometric analysis was performed using in-house software developed for MATLAB.

#### Hemodynamics

4D Flow data was spatially up-sampled using cubic interpolation and subsequently registered to the CE-MRA images to facilitate analysis using the segmentations generated by the CNN. Registration was performed using the Elastix registration toolbox (38, 39). Registrations were 3D affine transformations. Registrations were visually inspected and manually corrected if necessary.

WSS was calculated using a previously described method (19, 21, 22, 24). In short, this method determines the inward normal vector for each point on the luminal surface as well as the velocity at several points along that vector. After defining the velocity to be zero at the wall, and fitting a smoothed spline to the interpolated velocities, the slope of this curve can be calculated to generate the wall shear rate. Finally, multiplying by the shear-dependent viscosity, as defined by the Carreau–Yasuda model, the WSS vectors for each point along the wall are created (19, 21). In this study, three points along the inward normal vector were used, and the vector itself had a length of 3 mm (21, 22, 24). These WSS algorithm parameters fall within the optimum range as defined by Potters et al. (19) during algorithm validation.

tWSS was investigated based on measurements of near-wall TKE (nwTKE). nwTKE was calculated using a previously described method (20). In short, this method calculates the amount of TKE near the luminal surface using a spherical convolution kernel. In this study, the convolution kernel had a radius of 2.5 mm. The TKE near the vessel wall is related to the turbulent WSS, which is unable to be directly measured as a result of inadequate spatial and temporal resolution (20, 40). nwTKE can be used to discern regions with relatively lower or higher tWSS (20, 40).

WSS and nwTKE were calculated throughout the cardiac cycle. Descriptive statistics including the minimum, median, mean, standard deviation, and maximum values of WSS and nwTKE were extracted throughout the cardiac cycle, for each carotid branch. The same statistics were calculated for the time-averaged WSS (TAWSS) and time-averaged nwTKE (TAnwTKE), for each branch. The minimum and maximum values of WSS and nwTKE were computed as the 10% and 90% values, respectively, in order to reduce the impact of noise-related outliers. In addition, the oscillatory shear index (OSI) was calculated, for each branch (41). The TAWSS results for all vessels were pooled to find the threshold values for which 80% of the cumulative surface area (SA) was exposed, and termed the SA80 threshold (42, 43).

The Reynolds number was also calculated for each vessel, through time, using the estimated vessel diameter and vessel mean velocity at that timeframe. Blood was estimated to have a density of 1.060 kg/m^3^, and dynamic viscosity was estimated as 3.2 × 10^−3^ pa·s.

### Statistical Analysis

Pairwise analyses using the Spearman rank correlation coefficient, ρ, were used to examine the relationships between WSS, OSI, and nwTKE parameters. Pairwise analyses using the Spearman rank correlation coefficient were also used to examine the relationships between geometric descriptions of the carotid bifurcation and hemodynamic stress parameters. Spearman rank correlation coefficients are described using the following scale: ± [0, 0.2] very weak, ± [0.2, 0.4] weak, ± [0.4, 0.6] moderate, ± [0.6, 0.8] strong, and ± [0.8, 1] very strong. Cohort-wide descriptive statistics for hemodynamic stress parameters and geometric parameters are presented as mean ± standard deviation, unless otherwise stated. The coefficient of variation (CoV) was also used to describe the variation of a given parameter within the cohort. Two-sample *t*-tests were used to determine whether or not there were differences between hemodynamic parameters per branch using a significance level (α) of 0.05.

## Results

One hundred and seventy nine subjects (358 carotid bifurcations) were segmented using the CNN and included in all analyses after 66 subjects were excluded based on a visual quality-assessment of the CE-MRA volume and the 4D Flow volumes, failed registrations or segmentations, or other acquisition errors. The majority of exclusions were a result of poor contrast-timing in the CE-MRA volume. Basic clinical characteristics, and geometric descriptions of the carotid bifurcation for this cohort are listed in Table 1. Subjects typically had one plaque per side. Within the cohort, there was substantial variation with respect to vessel diameters. The diameter of the CCA showed the least variation of the branches, with a CoV of 12.6%, while the CoV was 17.5% and 18.6% in the ECA and ICA, respectively. The bifurcation angle had a CoV of 27% within the cohort. The cycle-average cohort-mean Reynolds numbers were 430 ± 171, 240 ± 110, and 280 ± 78 for the CCA, ECA, and ICA, respectively. Systolic cohort-mean Reynolds numbers were 821, 496, and 441 for the CCA, ECA, and ICA.

Several hemodynamic parameters are shown in Table 2, and a summary of the statistical tests is presented in Supplementary Table 1. The ECA had the highest TAWSS, followed by the CCA and the ICA. Values for maximum WSS and systolic WSS followed the same pattern. Similarly, the ECA had the highest TAnwTKE, maximum nwTKE, and systolic nwTKE. The OSI was lower in the ECA than the CCA, but no statistically significant differences were found when comparing the CCA and ICA, or the ECA and ICA.

**Table 2:** Cohort mean hemodynamic parameters (*n* = 358).

**Parameter**	**CCA**	**ECA**	**ICA**
TAWSS [Pa]	0.59 ± 0.19♦	0.64 ± 0.22♣♥	0.56± 0.18♦
Systolic WSS(t) [Pa]	1.08 ± 0.38♦♥	1.24 ± 0.46♣♥	0.86 ± 0.31♣♦
Maximum WSS(t) [Pa]	2.40 ± 0.74♦♥	2.79 ± 0.89♣♥	2.08 ± 0.63♣♦
OSI [-]	0.072 ± 0.036♦	0.067 ± 0.030♣	0.069 ± 0.029
TAnwTKE [J/m^3^]	8.42 ± 3.89♦	10.94 ± 4.36♣♥	7.91 ± 3.28♦
Maximum nwTKE(t) [J/m^3^]	18.30 ± 11.25♦♥	25.15 ± 14.36♣♥	13.91 ± 7.13♣♦
Systolic nwTKE(t) [J/m^3^]	39.06 ± 17.92♦♥	51.84 ± 26.73♣♥	32.60 ± 14.09♣♦

*All parameters are listed as mean ± sd*.

*♣ = statistically significant difference to CCA*.

*♦ = statistically significant difference to ECA*.

*♥ = statistically significant difference to ICA*.

*A full listing of p-values for all tests is given in Supplementary Table 1*.

**Figure 1:**
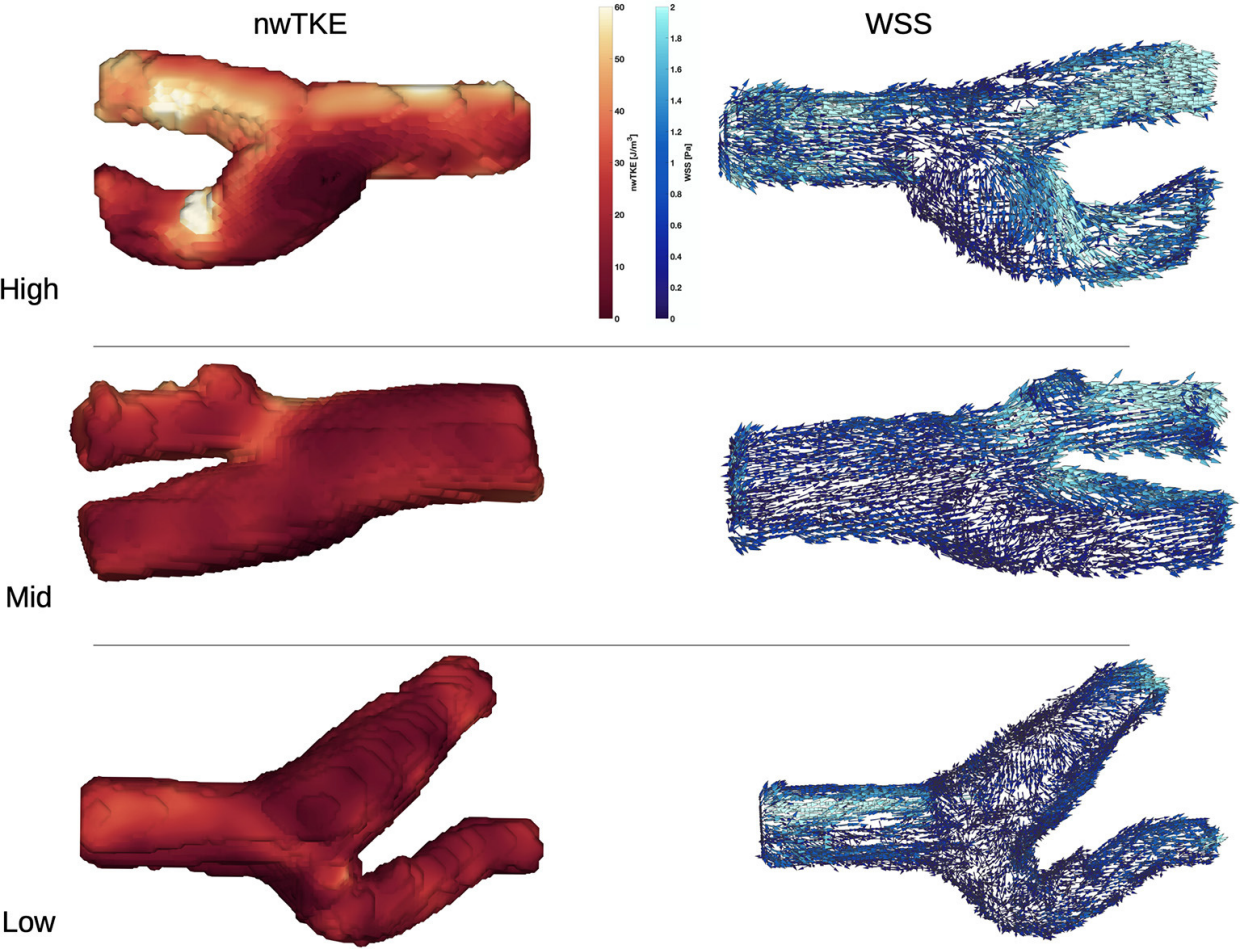
nwTKE **(Left)** and WSS **(Right)** depicted at peak systole e.g., bifurcations in the low, mid, and high tertiles. Views in the high and mid tertiles have been rotated by 180°.

**Figure 2:**
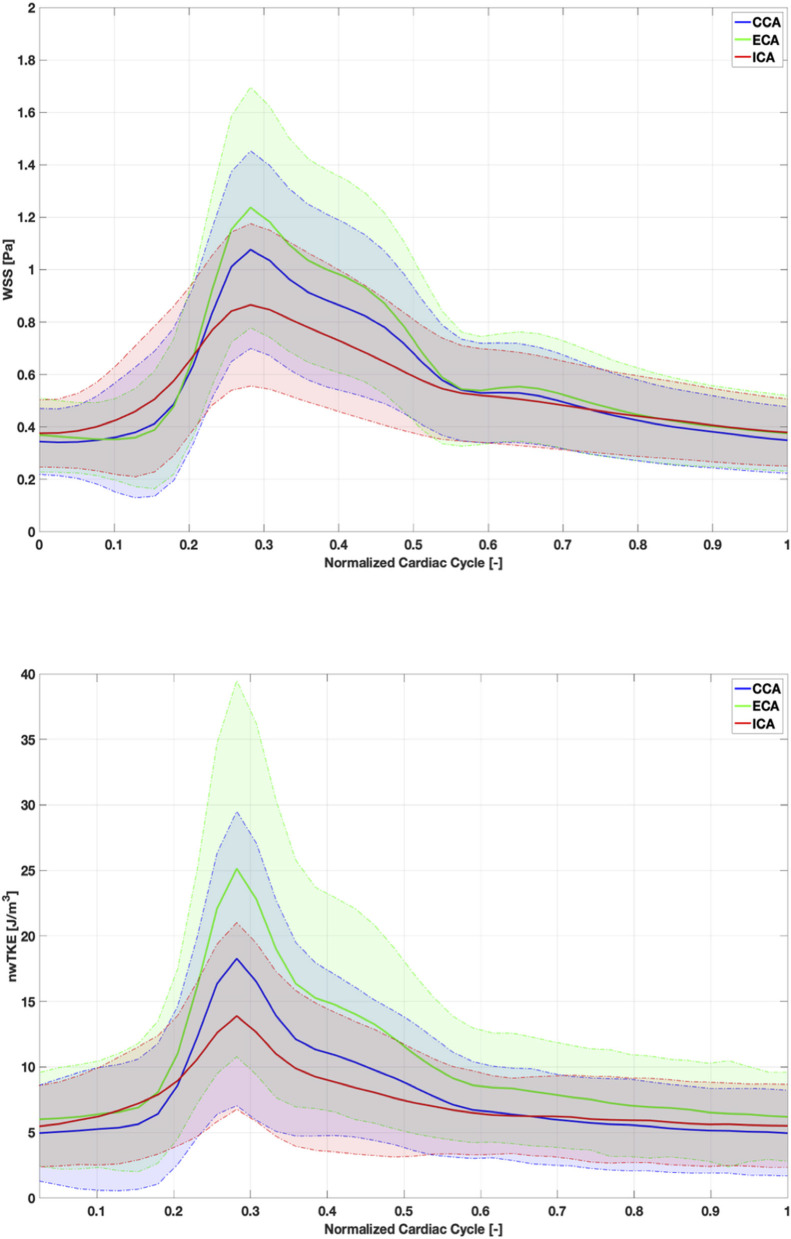
Cohort-mean and standard deviation of WSS **(top)** and nwTKE **(bottom)** through the cardiac cycle. Solid lines depict mean value and shaded area represents ±1 standard deviation.

WSS and nwTKE at peak systole for example subjects from the lower, mid, and high tertiles are shown in Figure 1. Figure 2 depicts the temporal evolution of the cohort mean and standard deviation values for both WSS(t) and nwTKE(t). The WSS and nwTKE in the three branches of the carotid artery follow similar waveforms. Differences between branches are minor during diastole, though statistically significant differences were found during systole (Table 2). The ECA was found to have the highest systolic WSS(t) and nwTKE(t). The ECA has the highest nwTKE throughout the cardiac cycle, as well as the largest standard deviation of nwTKE within the branch. Statistically significant differences were also found between the CCA and the ECA, and between the ECA and the ICA, when considering TAWSS and TAnwTKE (Table 2).

The SA80 threshold values for TAWSS in the CCA, ECA, and ICA were 0.66, 0.72, and 0.68 [Pa], respectively. There was considerable spread in the amount of vessel surface area exposed to TAWSS below these thresholds, resulting in coefficients of variation within the cohort of 60, 60, and 53% for the CCA, ECA, and ICA, respectively. This indicates large interindividual variations in TAWSS patterns.

The correlation coefficients for pairwise comparisons between the time-resolved parameters are listed in Table 3. Time-resolved WSS and nwTKE parameters tended to be moderately-to-strongly correlated. The weakest correlation was found when comparing maximum nwTKE(t) and minimum WSS(t).

**Table 3:** Spearman rank correlation coefficients for pairwise comparisons: time-resolved parameters.

	**nwTKE(t)**	**Max WSS(t)**	**Max nwTKE(t)**	**Min WSS(t)**	**Min nwTKE(t)**
**WSS(t)**	0.645	0.973	0.636	0.800	0.505
**nwTKE(t)**		0.633	0.910	0.493	0.842
**Max WSS(t)**			0.642	0.681	0.473
**Max nwTKE(t)**				0.432	0.670
**Min WSS(t)**					0.461
**Min nwTKE(t)**					

*All correlation coefficients were statistically significant (i.e. p ≤ 0.05). A full listing of p-values is given in Supplementary Table 2*.

**Figure 3:**
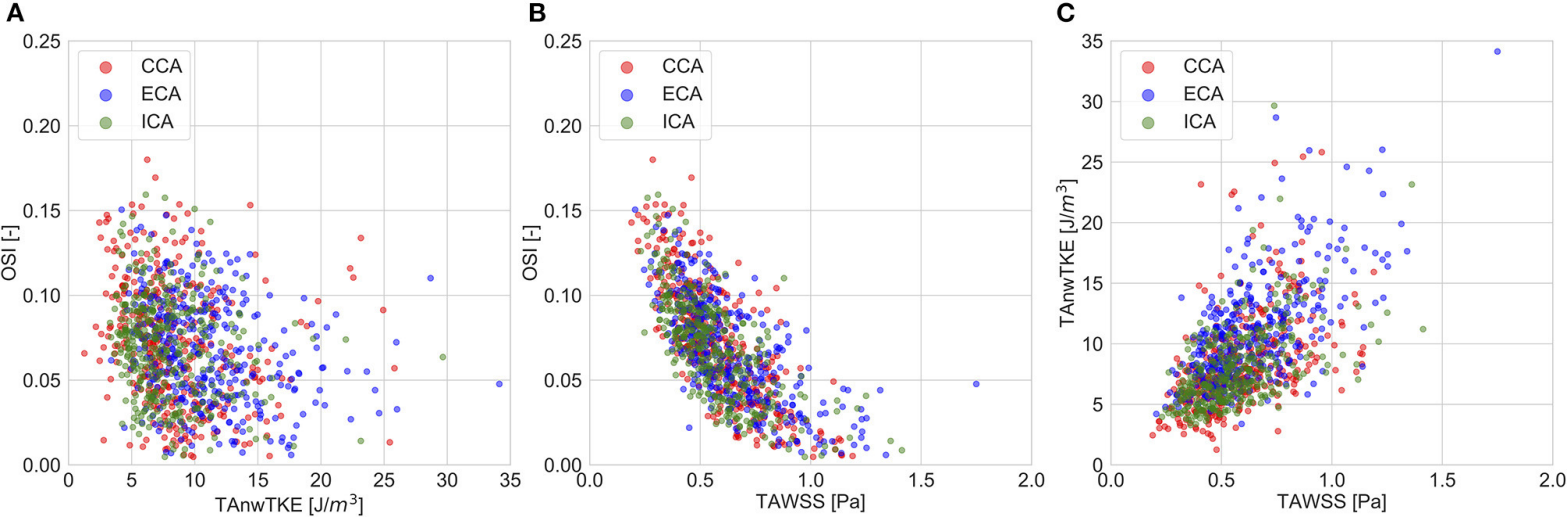
Scatter plots for selected pairwise comparisons of whole-cycle hemodynamic parameters. **(A)**—TAnwTKE vs. OSI; **(B)**—TAWSS vs. OSI; **(C)**—TAWSS vs. TAnwTKE.

The correlation coefficients for pairwise comparisons between the non-temporally resolved parameters are listed in Table 4. OSI, a commonly used measure for describing “disturbed” flow, was weakly related to TAnwTKE. Scatterplots for selected relationships are presented in Figure 3.

**Table 4:** Spearman rank correlation coefficients for pairwise comparisons: whole-cycle hemodynamic parameters.

	**TAnwTKE**	**OSI**	**SA80**	**Peak WSS(t)**	**Peak nwTKE(t)**
**TAWSS**	0.573	−0.751	0.933	0.843	0.464
**TAnwTKE**		−0.273	0.455	0.543	0.804
**OSI**			−0.690	−0.499	−0.184
**SA80**				0.778	0.356
**Peak WSS(t)**					0.587
**Peak nwTKE(t)**					

*All correlation coefficients were statistically significant (i.e. p ≤ 0.05). A full listing of p-values is given in Supplementary Table 3*.

**Figure 4:**
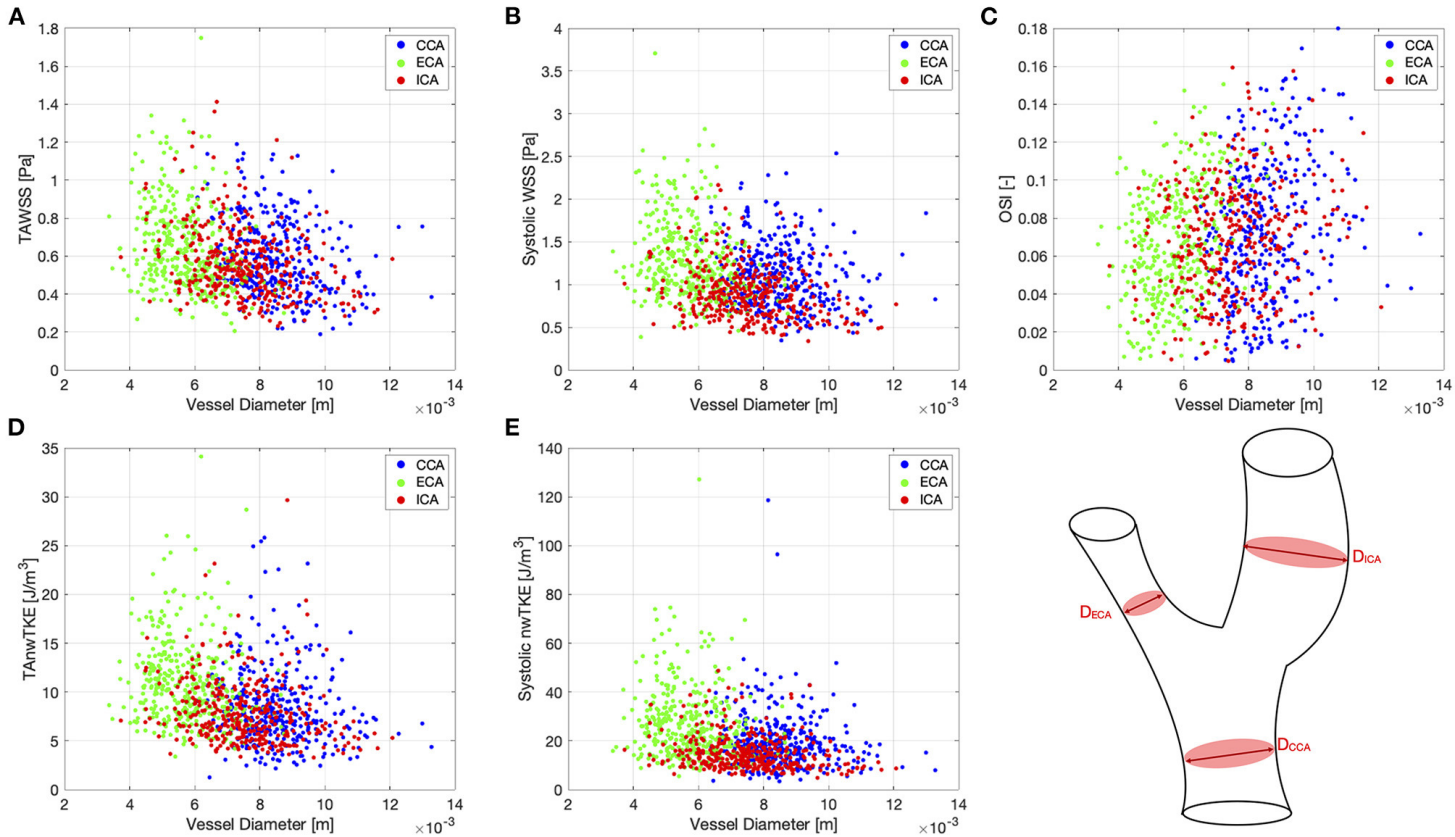
Scatter plots for pairwise comparisons between hemodynamic parameters and vessel diameter. **(A)**—TAWSS; **(B)**—Systolic WSS; **(C)**—OSI; **(D)**—TAnwTKE; **(E)**—Systolic nwTKE. Schematic diagram of carotid artery depicting the vessel diameters at lower right.

**Figure 5:**
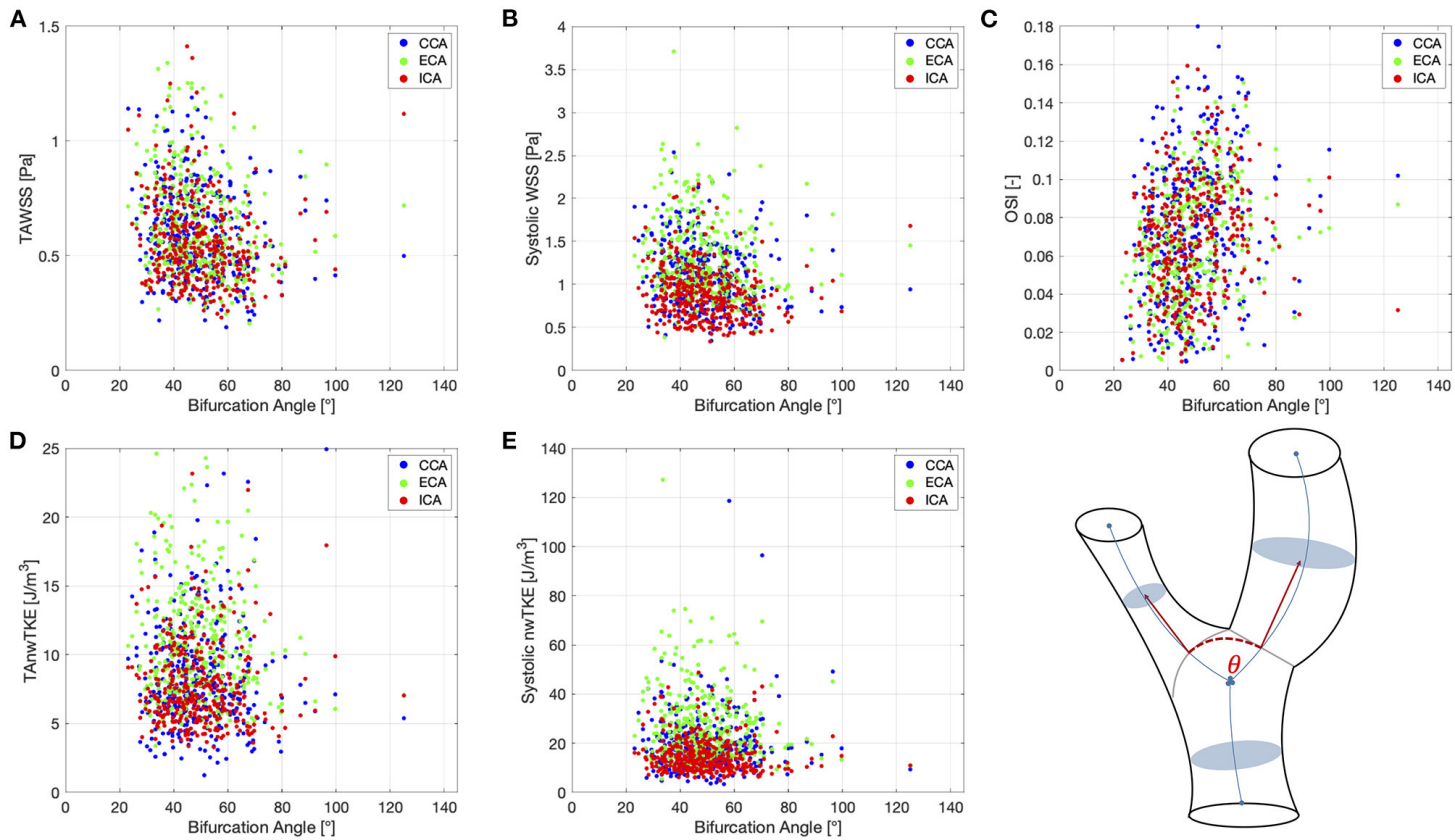
Scatter plots for pairwise comparisons between hemodynamic parameters and the bifurcation angle. **(A)**—TAWSS; **(B)**—Systolic WSS; **(C)**—OSI; **(D)**—TAnwTKE; **(E)**—Systolic nwTKE. Schematic diagram of carotid artery depicting the bifurcation angle at lower right.

Correlation coefficients for pairwise comparisons between selected hemodynamic parameters (TAWSS, TAnwTKE, systolic WSS, systolic nwTKE, and OSI) and geometric parameters (diameter, bifurcation angle) are listed in Table 5, and scatterplots for these assessments are shown in Figures 4, 5, respectively. Correlations were typically weak or very weak, and in general nwTKE had weaker correlations to the geometric parameters than WSS. The ICA and ECA tended to have stronger relationships than the CCA.

**Table 5:** Correlations between selected hemodynamic parameters and geometric parameters.

***hemodynamic parameter, y—geometric parameter, x***	**ρ**		***p***
TAWSS—Branch diameter	CCA	−0.2566	9.396e-07^*^
	ECA	−0.3029	5.886e-09^*^
	ICA	−0.3070	3.600e-09^*^
TAWSS—Bifurcation angle	CCA	−0.1950	0.0002^*^
	ECA	−0.2241	1.949e-05^*^
	ICA	−0.1883	0.0003^*^
TAnwTKE—Branch diameter	CCA	−0.1474	0.0052
	ECA	−0.1969	0.0001^*^
	ICA	−0.2537	1.250e-06^*^
TAnwTKE—Bifurcation angle	CCA	−0.0242	0.6471
	ECA	−0.0479	0.3655
	ICA	−0.0873	0.0988
Systolic WSS—Branch diameter	CCA	−0.1791	0.0006^*^
	ECA	−0.2691	2.613e-07^*^
	ICA	−0.2707	2.192e-07^*^
Systolic WSS—Bifurcation angle	CCA	−0.1808	0.0005^*^
	ECA	−0.2535	1.270e-06^*^
	ICA	−0.1966	0.0001^*^
Systolic nwTKE—Branch diameter	CCA	−0.1175	0.0261^*^
	ECA	−0.1519	0.0039^*^
	ICA	−0.2365	6.461e-06^*^
Systolic nwTKE—Bifurcation angle	CCA	0.0023	0.9640
	ECA	−0.1071	0.0427^*^
	ICA	−0.1325	0.0120^*^
OSI—Branch diameter	CCA	0.2158	3.992e-05^*^
	ECA	0.3226	5.112e-10^*^
	ICA	0.1729	0.0010^*^
OSI—Bifurcation angle	CCA	0.2257	1.698e-05^*^
	ECA	0.3329	1.325e-10^*^
	ICA	0.1966	0.0001^*^

* *p < 0.05*.

## Discussion

This study characterized and compared several different expressions of hemodynamic shear stresses acting upon the vessel wall in a cohort of 179 subjects with asymptomatic carotid atherosclerosis. This study also explored the relationship between these shear stresses and the geometry of the carotid bifurcation. In addition, nwTKE was for the first time investigated *in vivo*, and this study therefore offers a unique assessment of turbulent stresses on the vessel wall.

Our results add evidence from a large cohort to the growing body of literature concerning MR-estimated WSS in the carotid bifurcation (16, 18, 19, 21–23, 25–31) (Supplementary Table 4). While the rapid development of methods for estimating WSS from MR velocity data makes comparisons to previous studies difficult, TAWSS magnitude in this study was similar to studies previously published using the same methodology and calculation parameters for WSS estimation (19, 21–24, 44). For example, Cibis et al. reported similar branch-specific estimates for TAWSS from six healthy volunteers (21). In a later study from the same authors, Cibis et al. (24) compared their MRI-based results against CFD in both the CCA and the ICA of elderly subjects with asymptomatic carotid atherosclerosis. Our results, with subjects of similar age and atherosclerotic burden, though acquired with improved spatial and temporal resolution, fall between the estimates of TAWSS from MRI and CFD presented in that study (24). This is in accordance with previous work demonstrating that MR-based WSS estimates are typically lower than CFD-derived estimates, a result of poor spatial resolution (21, 22, 24). Finally, cohort average TAWSS values considering the entire bifurcation in this study (0.60 ± 0.20 Pa) are very similar to TAWSS values (0.56 ± 0.31 Pa) in the study by Van Ooij et al. (23). In that study, 40% of bifurcations studied had asymptomatic atherosclerotic plaques, while the remaining 60% were healthy. Bifurcation maximum WSS in our data (2.42 ± 0.75 Pa) is higher than the study by Van Ooij (1.85 ± 0.57) and this may be a consequence of our cohort being composed only of subjects with asymptomatic plaques and no healthy volunteers.

WSS parameters displayed substantial variation within the cohort in our data. For example, with respect to TAWSS, the coefficients of variation were 32, 34, and 32% in the CCA, ECA, and ICA. However, this is in line with previously reported data and supports the conclusion that the carotid bifurcation has complex and variable flow patterns. For example, in branch-specific data from healthy volunteers, TAWSS coefficients of variation of 28, 32, and 35% in the CCA, ECA, and ICA were reported by Cibis et al. (21). In a subsequent study from Cibis et al. (24), the coefficient of variation was 26% when considering the TAWSS of the entire carotid bifurcation in subjects with subjects of similar age and atherosclerotic burden.

Our data yielded a similar correlation between OSI and TAWSS as seen in previous CFD-based work (45), though values are lower. In examining the relationship between MRI- and CFD-based estimates of OSI in the carotid bifurcation, Cibis et al. found a significant association between OSI and spatial resolution, where OSI decreased as spatial resolution worsened (22). It has been shown that MRI tends to underestimate peak WSS values when compared to CFD, and has decreased measurement accuracy at very low velocities as a result of lower SNR. These factors likely account for the lower OSI found in our data when compared to CFD data with higher spatial and temporal resolution (22, 44, 45).

In this study, nwTKE and WSS were examined together for the first time *in vivo*. Temporally resolved nwTKE and WSS were strongly correlated, ρ = 0.645, which can be expected as turbulence at the vessel wall to some degree develops near regions with high velocity and resulting flow detachment. The maximum nwTKE and maximum WSS values per branch were more strongly correlated (ρ = 0.642) than the minimum pairing (ρ = 0.460) at a given point in time. As visualized in Figure 2, peak nwTKE tends to occur following peak WSS as the flow decelerates. While we have examined the relationship between these parameters in a large cohort of subjects with asymptomatic atherosclerosis, examinations of subjects with more severe stenoses are of interest, as they may yield a different relationship between nwTKE and WSS given that the amount of turbulence present in the flow increases dramatically.

nwTKE provides information about the impact of disturbed flow on the vessel wall that is complementary to conventional assessment using OSI. OSI has been used as an indirect measurement for the effects of disturbed flow on the vessel wall as it measures the degree of flow reversal. While OSI represents laminar and periodic oscillations in WSS over the cardiac cycle, nwTKE represents rapid chaotic oscillations in WSS on the order of milliseconds or less. Indeed, we did not find strong correlations between OSI and TAnwTKE, in any branch, in our analyses. Similarly, TAnwTKE was not correlated to SA80, as expected given the strong relationship between SA80 and OSI (ρ = −0.690). This signals that nwTKE presents information that OSI does not, and therefore both should be examined to gain a more complete understanding of the stresses acting on the vessel wall.

Vessel diameter and bifurcation angle displayed, at best, weak correlations to hemodynamic parameters in this study. Three whole-cycle parameters were examined: TAWSS, TAnwTKE, and OSI, as well as systolic WSS and systolic TKE. The strongest correlations were found between branch diameter and TAWSS, though these were still weak, and between OSI and the geometric parameters in the ECA. Our results therefore do not point to branch diameter or bifurcation angle alone as obvious or robust variables for predicting WSS or nwTKE, and add to previous research with similar findings (46). More complex regression models, in the spirit of those proposed by Lee et al. (42) could be examined, and studies with subjects spanning the range from healthy to severely atherosclerotic are therefore warranted in more exhaustive examinations of carotid bifurcation geometry and its implications for the atherosclerosis geometric risk hypothesis.

### Limitations

This study has several limitations. It is well-known that MR-based WSS underestimates the true WSS due to poor spatial resolution. In addition, MRI-based measurements are subject to noise and segmentation errors that affect WSS estimates (19, 22, 44, 47). A comparison against CFD for the purposes of an error propagation analysis was not performed, though previous studies using the same WSS calculation methodology can illuminate this area (19, 21, 22). Despite these known limitations 4D Flow MRI-derived shear stress parameters have been determined to show reasonably accurate distributions and are comparable between studies with similar acquisition and calculation parameters (44). Similarly, a reproducibility analysis of the segmentation and registration methods used in this study was not performed, but is a subject of future investigations. However, recent research has shown that WSS studies have low variability even when using a semi-automatic segmentation method, as opposed to the automated method in this study (48). This study focused on the characterization and comparison of different expressions of shear stress in subjects with carotid atherosclerosis. Unfortunately, no cohort of subjects without atherosclerosis was available and therefore no comparisons against control subjects could be made, though this needs to be explored in future studies to establish values of abnormal WSS and nwTKE. Future studies with a control cohort would also have the ability to examine the relationships between geometric and hemodynamic parameters in a more exhaustive manner. Additionally, this study did not include follow-up examinations and therefore the clinical implications of the different expressions of shear stress studied here need to be investigated further in longitudinal studies.

## Conclusion

In conclusion, this study estimated the hemodynamic wall stress parameters WSS, OSI, and nwTKE in a cohort of 358 carotid bifurcations and investigated the relationships between them as well as their relationships to basic geometric parameters. Correlations between WSS and nwTKE were moderate-to-strong for both their temporally resolved and cycle-averaged forms. In addition, nwTKE, reflecting the turbulent component of WSS, was not related to commonly used parameters for estimating the effects of disturbed flow such as the OSI and therefore appears to present new information. Hemodynamic parameters displayed weak correlations to vessel diameter and the bifurcation angle. WSS, OSI, and nwTKE exhibited substantial variation throughout the cohort, indicating that the degree and pattern of hemodynamic stresses acting on the vessel wall are not easily predicted and should be revealed using comprehensive *in vivo* investigations.

## Data Availability Statement

The raw data supporting the conclusions of this article will be made available by the authors, without undue reservation.

## Ethics Statement

This study was reviewed and approved by the Ethical Review Board at Umeå University (Umeå, Sweden, diary number 2010-228-31M). Participants provided their written informed consent to participate in this study.

## Author Contributions

MZ, EdM, and PD conceived and designed the study. MZ, JA, EG, and PD analyzed the data. MZ, EG, JE, EdM, and PD interpreted the results. MZ and PD drafted the manuscript. All authors reviewed and approved the final manuscript.

## Conflict of Interest

The authors declare that the research was conducted in the absence of any commercial or financial relationships that could be construed as a potential conflict of interest.
