# Erratum: Exploring the Relationships Between Hemodynamic Stresses in the Carotid Arteries

**DOI:** 10.3389/fcvm.2021.669888

**Published:** 2021-03-11

**Authors:** Frontiers Production Office

**Affiliations:** Frontiers Media SA, Lausanne, Switzerland

**Keywords:** carotid bifurcation, magnetic resonnance imaging (MRI), turbulence, wall shear stress (WSS), atherosclerosis

Due to a production error, there was a mistake in **the author affiliations** as published. The correct affiliations appear below:

Magnus Ziegler^1,2^^*^, Jesper Alfraeus^1,2^, Elin Good^1,2,3^, Jan Engvall^1,2,4^, Ebo de Muinck^1,2,3^ and Petter Dyverfeldt^1,2^

1 Division of Cardiovascular Medicine, Department of Health, Medicine and Caring Sciences, Linköping University, Linköping, Sweden

2 Center for Medical Image Science and Visualization (CMIV), Linköping University, Linköping, Sweden

3 Department of Cardiology, Department of Health, Medicine and Caring Sciences, Linköping University, Linköping, Sweden

4 Department of Clinical Physiology, Department of Health, Medicine and Caring Sciences, Linköping University, Linköping, Sweden

*Correspondence: Magnus Ziegler, magnus.ziegler@liu.se

The publisher apologizes for these mistakes. The original article has been updated.

