# Supplementary material for: Exploring the Relationships Between Hemodynamic Stresses in the Carotid Arteries
Source: Front Cardiovasc Med. 2021 Feb 3;7:617755. doi: 10.3389/fcvm.2020.617755 (PMC7886794; doi:10.3389/fcvm.2020.617755)
Supplement: Supplementary file 1 [file Data_Sheet_1.docx]

**Supplementary Table 1: Summary of Statistical Tests**

| **Parameter** | **Test Pair** | **Mean Difference** | **p-value** |
| --- | --- | --- | --- |
| TAWSS [Pa] | CCA vs. ECA* | -0.0524 | 8.2299e-04 |
|  | CCA vs. ICA | 0.0224 | 0.1179 |
|  | ECA vs. ICA* | 0.0748 | 1.3542e-06 |
| Systolic WSS [Pa] | CCA vs. ECA* | -0.1834 | 8.4133e-09 |
|  | CCA vs. ICA* | 0.2068 | 4.9727e-15 |
|  | ECA vs. ICA* | 0.3901 | 7.9328e-36 |
| Maximum WSS [Pa] | CCA vs. ECA* | -0.3876 | 4.1144e-10 |
|  | CCA vs. ICA* | 0.3139 | 1.7751e-09 |
|  | ECA vs. ICA* | 0.7015 | 1.3379e-30 |
| OSI [-] | CCA vs. ECA* | 0.0053 | 0.0342 |
|  | CCA vs. ICA | 0.0029 | 0.2427 |
|  | ECA vs. ICA | -0.0024 | 0.2855 |
| TAnwTKE [J/m^3^] | CCA vs. ECA* | -2.5225 | 1.6103e-15 |
|  | CCA vs. ICA | 0.5092 | 0.0592 |
|  | ECA vs. ICA* | 3.0317 | 5.9627e-24 |
| Systolic nwTKE [J/m^3^] | CCA vs. ECA* | -7.4179 | 5.0676e-14 |
|  | CCA vs. ICA* | 4.6481 | 8.8320e-11 |
|  | ECA vs. ICA* | 12.0660 | 4.1000e-39 |
| Maximum nwTKE [J/m^3^] | CCA vs. ECA* | -12.7855 | 1.9680e-13 |
|  | CCA vs. ICA* | 6.4543 | 1.1712e-07 |
|  | ECA vs. ICA* | 19.2398 | 8.8777e-30 |

*- p < 0.05

**Supplementary Table 2: Correlation Statistics – Time-Resolved Hemodynamic Parameters**

| **Test Pair** | **Spearman’s rho** | **p** |
| --- | --- | --- |
| WSS(t) – nwTKE(t) | 0.645 | <1e-20 |
| WSS(t) – Max WSS(t) | 0.973 | <1e-20 |
| WSS(t) – Max nwTKE(t) | 0.636 | <1e-20 |
| WSS(t) – Min WSS(t) | 0.800 | <1e-20 |
| WSS(t) – Min nwTKE(t) | 0.505 | <1e-20 |
| nwTKE(t) – Max WSS(t) | 0.633 | <1e-20 |
| nwTKE(t) – Max nwTKE(t) | 0.910 | <1e-20 |
| nwTKE(t) – Min WSS(t) | 0.493 | <1e-20 |
| nwTKE(t) – Min nwTKE(T) | 0.842 | <1e-20 |
| Max WSS(t) – Max nwTKE(t) | 0.642 | <1e-20 |
| Max WSS(t) – Min WSS(t) | 0.681 | <1e-20 |
| Max WSS(t) – Min nwTKE(t) | 0.473 | <1e-20 |
| Max nwTKE(t) – Min WSS(t) | 0.432 | <1e-20 |
| Max nwTKE(t) – Min nwTKE(t) | 0.670 | <1e-20 |
| Min WSS(t) – Min nwTKE(t) | 0.461 | <1e-20 |

**Supplementary Table 3: Correlation Statistics – Whole-Cycle Hemodynamic Parameters**

| **Test Pair** | **Spearman’s rho** | **p** |
| --- | --- | --- |
| TAWSS – TAnwTKE | 0.573 | <1e-20 |
| TAWSS – OSI | -0.751 | <1e-20 |
| TAWSS – SA80 | 0.933 | <1e-20 |
| TAWSS – Peak WSS(t) | 0.843 | <1e-20 |
| TAWSS – Peak nwTKE(t) | 0.464 | <1e-20 |
| TAnwTKE – OSI | -0.273 | 1.2e-19 |
| TAnwTKE – SA80 | 0.455 | <1e-20 |
| TAnwTKE – Peak WSS(t) | 0.543 | <1e-20 |
| TAnwTKE – Peak nwTKE(t) | 0.804 | <1e-20 |
| OSI – SA80 | -0.690 | <1e-20 |
| OSI – Peak WSS(t) | -0.499 | <1e-20 |
| OSI – Peak nwTKE(t) | -0.184 | 1.4e-9 |
| SA80 – Peak WSS(t) | 0.778 | <1e-20 |
| SA80 – Peak nwTKE(t) | 0.356 | <1e-20 |
| Peak WSS(t) – Peak nwTKE(t) | 0.587 | <1e-20 |

| **Supplementary Table 4: Previous WSS studies in the Carotid Bifurcation** | | | |
| --- | --- | --- | --- |
| **Author, Journal, Year** | **Data** | **WSS [Pa]** | **Notes** |
| van Ooij, Eur.Radio.Exp, 2018 | - 20 Bifurcations - 40% with asymptomatic plaques | TAWSS: 0.56 ± 0.31  Peak: 1.85 ± 0.57 | - values via author correspondence  - 3D  - Potters Method  - 4D Flow MRI  - 3.5 cP Carreau-Yasuda |
| Cibis, J.Biomech, 2016 | - 14 Bifurcations - Asymptomatic  (min 2.5mm plaques) | 0.50 ± 0.13 | - 3D  - Potters Method  - 4D Flow MRI - 3.5 cP Carreau-Yasuda |
| Cibis, NMR Biomed, 2014 | - 9 healthy bifurcations | 0.62 ± 0.18 (cohort, whole vessel)  CCA: 0.60±0.17  ECA: 0.56±0.18  ICA: 0.73±0.26 | - 3D  - Potters Method  - 4D Flow MRI  - 3.5 cP Carreau-Yasuda |
| Potters, JMRI, 2014 | - 3 healthy, young bifurcations | 1.34 ± 0.72, whole CA TAWSS | - First 3D approach in carotid  - 4D Flow MRI - 3.2 cP |
| Harloff, Eur. Radiology, 2013 | - 20 Bifurcations, - 75% with symptomatic ICA stenosis. -Pre + Post CEA surgery | CCA: pre 0.49±0.20,  post 0.47±0.32  ICA: pre 0.72±0.30,  post 0.45±0.21  ECA: pre 0.87±0.45,  post 0.87±0.73 | - 2D planes  - 4D Flow MRI  - no viscosity stated |
| Markl, Circ: Cardiovasc. Img, 2010 | - 64 healthy, young bifurcations  - 6 older with moderate stenosis (48 ± 6%) in ICA - 11 older with recanalized former severe ICA stenosis (85 ± 9%) | 0.5* * - No direct numbers given for TAWSS. Value is an estimate from figures | - 2D planes  - 4D Flow MRI  - no viscosity stated |
| Sui, Acta Radiologica, 2009 | - 28 healthy, young ICAs | 0.56 ± 0.18 (mean) 0.84 ± 0.31 (peak systole) | - 2D PC-MRI  - Oyre Method  - 3.5 cP |
| Box, JMRI, 2007 | - 27 healthy, young ICAs | 1.67 ± 0.33 (systolic) | - 2D PC-MRI - 4.6 cP |
| Gelfand, JMRI, 2006 | - 6 healthy, young CCA + ICA Sinus (ICS) | CCA: 1.15 ± 0.15 (mean),  3.34 ± 0.41 (max),  0.3±0.16 (min) ICS: 0.13 ± 0.08 (mean),  0.95 ± 0.17 (max),  -0.71±0.19 (min) | - 2D PC-MRI - 4.0 cP |
| Oshinski, JCMR, 2006 | - 16 bifurcations with bilateral atherosclerosis - 8 healthy | 0.8 ± 0.41 (healthy)  0.75 ± 0.25 (patients) | - 2D PC-MRI  - 4.0 cP |
| Stokholm, Eur.J.Vasc.Endovasc.Surg, 2004 | - 8 healthy, young bifurcations at flow divider and lateral wall of bifurcation | 3.10 ± 0.67 (flow divider)  0.24 ± 0.16 (lateral wall) | - 2D PC-MRI  - 4.0 cP |
| Wu, Mag. Res. Img., 2004 | - 10 healthy, young bifurcations at 1.4 cm upstream from flow divider | 1.02 ± 0.22 (mean) 2.0 ± 0.53 (max/sys) 0.14 ± 0.27 (min/dia) | - 2D PC-MRI  - no viscosity stated |
| Wu, JMRI, 2004 | - 20 healthy, young bifurcations  3 cm upstream from flow divider | 1.17 ± 0.21 (mean)* 3.32 ± 0.43 (max/sys)* 0.41 ± 0.38 (min/dia)* | - 2D PC-MRI  * = Wall shear rates given in article, WSS estimated here using 3.5 cP |
| Oyre, J. Am. Cardiol, 1998 | - 7 healthy, young bifurcations  2 cm below bifurcation | 0.95 ± 0.04 (mean), 2.56 ± 0.08 (peak) | - 2D PC-MRI  - 4.3 cP - 3DP = 3D paraboloid method |
